# 2409: Opportunities and challenges for precision medicine and biomarkers: A regulatory science case study

**DOI:** 10.1017/cts.2017.143

**Published:** 2018-05-10

**Authors:** Steven Murdy, Scott J. Steele, Joan E. Adamo

## Abstract

OBJECTIVES/SPECIFIC AIMS: To develop a regulatory science case study as an educational resource to inform the regulatory science considerations in medical product development for a range of scientific priority areas and emerging technologies. METHODS/STUDY POPULATION: Precision medicine represents one of the major regulatory science priority areas and the use of biomarkers holds promise for predicting the response to individual treatment strategies. Although progress has been made toward developing biomarkers, the development and validation of clinically useful biomarkers has presented significant regulatory science challenges, including the utilization of biomarkers in predicting responses to different cancer therapies. This case study reviews the technical, regulatory, and policy issues related to the development and use of lung cancer drugs Opdivo^®^ and Keytruda^®^ and an understanding of the codevelopment and utilization of their associated biomarkers. RESULTS/ANTICIPATED RESULTS: A detailed instructor guide with extensive resources such as diagrams and timelines will accompany the case study and will be used to highlight the development and approval process of 2 competing drugs and their associated biomarkers. The resources will provide a better understanding of their progression through the FDA regulatory process and opportunities and challenges for their use. DISCUSSION/SIGNIFICANCE OF IMPACT: Building on the case study framework we have developed, the detailed timelines and a collection of available resources, an extensive and modular case study will be finalized and made available to academic institutions, industry, regulatory agencies, and the public. The full case study and links to a series of resources will be disseminated as a standalone resource for integration into courses or programs interested in learning about specific regulatory science needs and opportunities to enhance medical product development and approval.

